# Exploration and prospect of core research hotspots in sepsis-induced myocardial injury based on bibliometrics

**DOI:** 10.1097/MD.0000000000050014

**Published:** 2026-07-31

**Authors:** Zhikang Mei, Menglu Zhang, Tao Hao, Zhirui Zhang, Hong Jiang

**Affiliations:** a Department of Colorectal Hernia Surgery, Shandong Medical and Pharmaceutical University Hospital, Binzhou, Shandong, China; b Department of Otorhinolaryngology, Head and Neck Surgery, Shandong Medical and Pharmaceutical University Hospital, Binzhou Medical University, Binzhou, Shandong, China.

**Keywords:** bibliometrics, CiteSpace, myocardial injury, sepsis, VOSviewer

## Abstract

**Objective:**

Sepsis-associated myocardial injury (SICM) is one of the most common and severe complications of sepsis. The present study aimed to analyze research trends, collaborative networks, and knowledge dissemination in SICM over the past decade using bibliometric methods, thereby providing a reference for future research directions.

**Method:**

Relevant literature published between 2015 and 2025 was retrieved from the Web of Science Core Collection (WOSCC) database. A bibliometric cross-sectional analysis was performed using software tools, including VOSviewer and CiteSpace, with corresponding evaluation metrics extracted or calculated. Publications were categorized by country, institution, author, journal, highly cited papers, and keywords; these variables were compared with respect to publication output and academic impact, followed by bibliometric and visual analyses.

**Results:**

Over the past decade (2015–2025), remarkable advances have been achieved in sepsis research related to SICM. A total of 2927 papers were included in this study. China, the United States, and England constituted the primary sources of publications in this field; among the top 20 contributing institutions, 15 were from China, 4 from the United States, and one from Australia, with Yang Yang identified as the core contributing author. Shock ranked first in the number of publications, while Critical Care topped the list in terms of citation frequency. The most frequently used keywords were sepsis, mortality, and septic shock. Analysis of keyword bursts revealed that precision regulation based on molecular mechanisms and the exploration of intervention targets represent the current research hotspots.

**Conclusion:**

Research on sepsis-associated SICM is flourishing. The present study clarifies the research hotspots in this field, provides a comprehensive overview of relevant research trends, and offers a reference for potential collaborations and future research directions.

## 1. Introduction

Sepsis is a systemic inflammatory response syndrome and also one of the leading causes of death in critically ill patients.^[1]^ As a severe complication of sepsis, myocardial injury can significantly increase the morbidity and mortality of patients.^[2]^ According to statistics, more than 50% of sepsis patients develop varying degrees of cardiac dysfunction in the early stage of the disease; the mortality rate of sepsis patients without myocardial injury is 20%, while that of patients complicated with myocardial injury is as high as 70% to 90%.^[3]^

In recent years, research on sepsis-associated myocardial injury (SICM) has advanced from the macroscopic pathological level to the mechanisms of molecular modification and immune regulation.^[4]^ Diagnostic approaches are moving toward early sensitization, and treatment strategies are focusing on individualized, targeted interventions.^[5]^ At present, a large amount of research literature on SICM has been published at home and abroad, mainly covering core fields such as epidemiological characteristics and pathogenic mechanism analysis.^[6]^ Despite the substantial increase in the number of studies, the pathogenesis of SICM remains incompletely elucidated. Meanwhile, the field still faces key bottlenecks, including inconsistent definitions, difficulties translating basic mechanistic research into clinical practice, and a lack of precise treatment regimens.^[7]^ Therefore, a systematic analysis of the research status, key areas, and prospects of SICM is of great significance for improving the understanding of this disease among clinical and scientific researchers.^[8]^

The bibliometric method has been widely used to explore the scientific research productivity of countries, institutions, and researchers in specific disciplines.^[9]^ It can also accurately identify significant research achievements, key events, and future development trends in the field. Through bibliometric analysis, it is possible to systematically evaluate the quality of publications and the academic performance of researchers in the field.^[10]^ This method can not only help experts in the field sort out the research context but also assist novices in quickly grasping the breadth of the discipline, exploring research hotspots, and clarifying future research plans in a visualized form.

With the explosive growth in the volume of scientific literature and the increasing importance of research impact evaluation, the role of bibliometrics in scientific research evaluation has become increasingly prominent.^[11]^ Its unique ability to predict potential research directions and development trajectories has made it an indispensable research tool. Thus, bibliometrics has been widely applied in various disciplines and is recognized as an essential means of scientific research evaluation.

## 2. Materials and methods

### 2.1. Data sources and search strategy

As one of the most essential and globally recognized databases, the Web of Science Core Collection (WoSCC) comprehensively covers more than 12,000 academic journals. It has been widely adopted by researchers as the preferred database for bibliometric analysis.^[12]^ The use of WoSCC enables more comprehensive literature retrieval and ensures the high-quality of included academic publications; additionally, the data retrieved from this database contain complete citation information, which facilitates subsequent knowledge graph analysis.^[13]^

**Figure 1.**
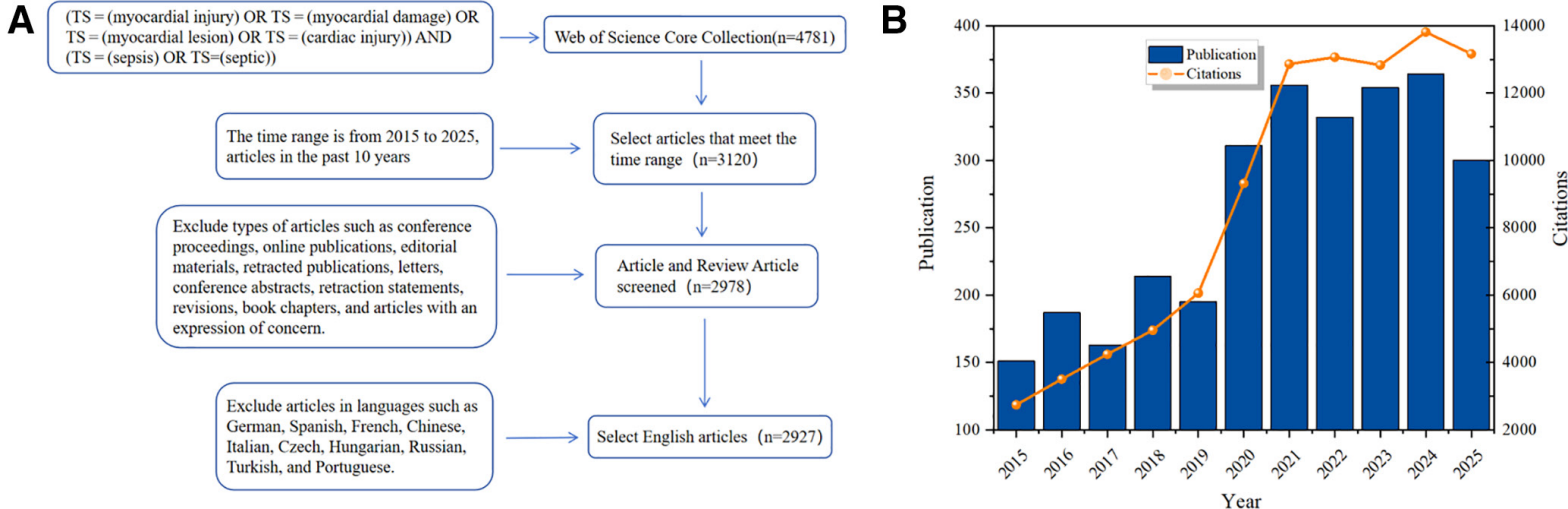
Retrieved data. (A) Literature search flow chart. (B) Publication output and trends.

After consultation with senior literature retrieval experts, all authors reached a consensus and finalized the search strategy. Subsequently, the WoSCC database was used to retrieve SICM-related literature published from January 1, 2015, to November 10, 2025. Inclusion criteria: literature types were reviews or original articles; written in English; containing complete information, including authors, titles, source journals, affiliated institutions, publication years, citations, keywords, research fields, and references; and published from January 1, 2015, to November 10, 2025. Exclusion criteria: conference papers, online preprints, editorials, retracted articles, letters, etc; non-English literature. Search formula: (TS = (myocardial injury) OR TS = (myocardial damage) OR TS = (myocardial lesion) OR TS = (cardiac injury)) AND (TS = (sepsis) OR TS = (septic)).^[14]^ The retrieved literature was exported as complete records and references in plain text format, then imported into EndNote X9 for duplicate removal. A total of 2927 articles were finally obtained. All retrieval operations were completed on the same day to avoid deviations in the number of literature records caused by real-time database updates. The detailed screening process is shown in Figure 1A.^[15]^

### 2.2. Data analysis and software application

Two researchers independently performed data extraction, literature screening, and analysis to ensure the reliability of the study results. First, Microsoft Excel was used for data preprocessing, followed by the import of all literature data into VOSviewer (Version 1.6.20) and CiteSpace (Version 6.2.R4). Meanwhile, the bibliometrix package in RStudio was used for data analysis and visualization.^[16]^ Information, including the h-index, impact factor (IF), and journal classification, was extracted from the WoSCC database.^[17]^ Finally, all data and charts were reviewed to correct duplicates and spelling errors, with the analysis focusing on the characteristics and impacts of co-authorship, co-occurrence, and co-citation in this field.

This study employed 3 tools: VOSviewer, CiteSpace, and Bibliometrix to conduct a bibliometric analysis of literature on SICM over the past decade. Each of these tools has unique advantages that complement the bibliometric analysis process, as detailed below. First, VOSviewer is one of the most widely used tools in bibliometric analysis.^[18]^ Developed by Nees Jan van Eck and Ludo Waltman in 2009, it is free software for constructing and visualizing bibliometric maps. It has distinct advantages for clearly presenting large-scale bibliometric maps. Secondly, CiteSpace is a fully functional visualization tool developed by Chen et al It provides a platform for visualizing co-occurrence networks in specific fields, accurately detecting research hotspots and predicting future research directions, making it a mainstream tool in current bibliometric research. Thirdly, Bibliometrix is an open-source R package. Its online platform (https://bibliometric.com/) relies on the R package and is primarily used for scientific mapping and graphical analysis in bibliometrics.^[19]^

## 3. Results

### 3.1. Publication trend analysis

A total of 2927 publications were retrieved in this study, involving 16,835 authors from 3846 institutions across 100 countries, and published in 200 journals with 109,514 cited references from 9241 journals. The annual publication output and citation trends are presented in Figure 1B.^[20]^ The number of publications grew rapidly from 2015 to 2020, increasing from 151 to 311. From 2021 to 2024, the field entered a high-level, stable phase, with publications remaining relatively stable at 332 to 364 per year. Further analysis revealed 3 distinct phases of development: the steady accumulation phase (2015–2019), during which the number of publications increased from 151 to 195 articles (average annual growth rate: 7.2%) and cumulative citations rose from 2744 to 6059 (average annual growth rate: 21.5%); the critical outbreak phase (2020), when publications surged to 311 articles (a 59.5% increase) and cumulative citations climbed to 9321 (a 53.8% increase); and the mature and stable phase (2021–2024), in which publication output stabilized between 330 and 364 articles (peaking at 364 in 2024) and cumulative citations remained above 12,800 (peaking at 13,813 in 2024).

In 2025, both the number of publications (300 articles) and cumulative citations (13,166) declined slightly. Given that 2025 has not yet concluded, this phenomenon is attributed to a stage-specific fluctuation caused by the data cutoff, and subsequent data are expected to rebound as more research findings are published.

### 3.2. Country and institution analysis

**Figure 2.**
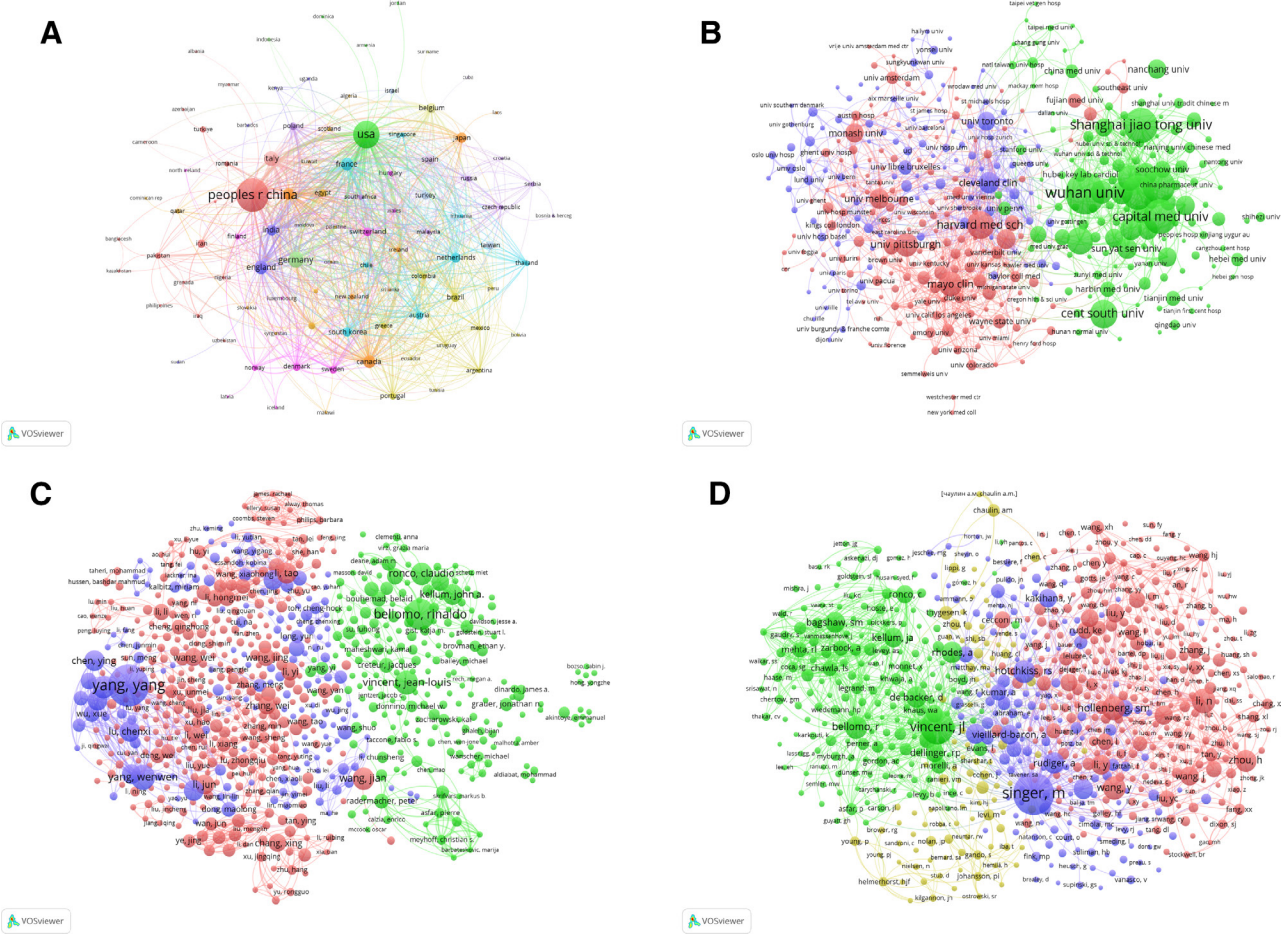
Collaboration network: (A) Country level, (B) Institution level, (C) Author level, (D) Journal level.

**Figure 3.**
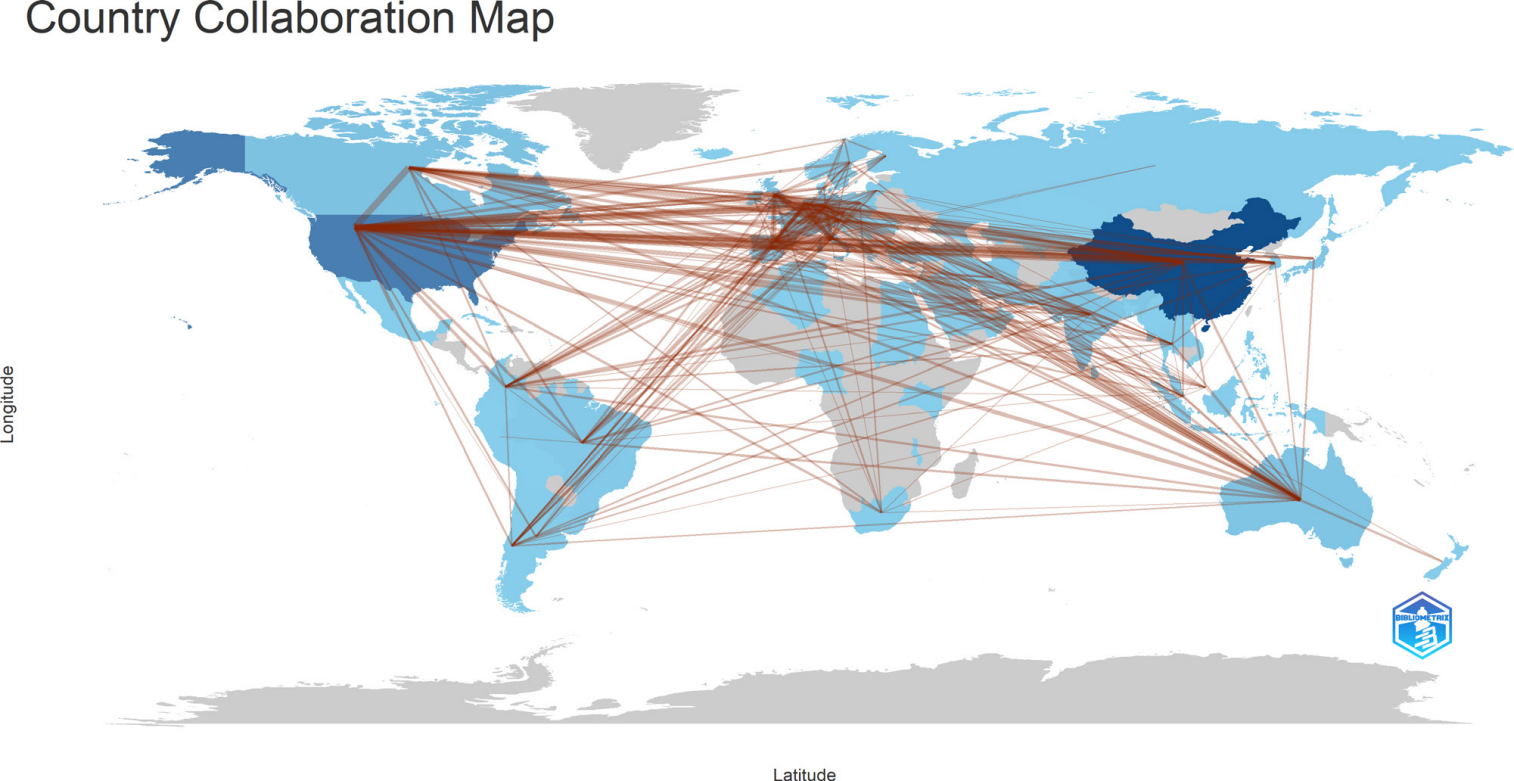
Country collaboration map.

The top 10 countries in terms of research productivity are presented in Table 1 and Figure 2A. Table 1 shows the total citations (TC) and citations per paper for these top 10 countries.^[21]^ China demonstrated outstanding performance, ranking first globally in both metrics with 1212 publications and 9331 TC. In terms of publication output, other major contributors included the United States (747 publications), Germany (187 publications), and Italy (166 publications), each achieving remarkable research outcomes. However, China’s citations per paper stood at 21.55, ranking only 51st worldwide and last among the top 10 countries. The top 3 countries in terms of citations per paper were England (54.47), Canada (48.51), and Germany (43.03). Notably, although Cameroon published only 2 articles, it ranked first globally in citations per paper, with 1128 TC and an average of 564 per paper. Figure 3 illustrates the collaborative relationships among countries: the United States, China, and European countries had the densest collaborative links, serving as the core entities of international cooperation.^[22]^ Specifically, the United States and China maintained extensive collaborations spanning Europe, South America, and Oceania, with a geographically dispersed cooperative network.

**Table 1:** Top 10 countries/regions and organizations related to septic myocardial injury.

Type	Rank	Country	Publications	TC	TC/publication	Organization	Publications	TC	TC/publication
Septic myocardial injury	1	Peoples R China	1212	26,117	21.55	Wuhan Univ	70	3174	45.34
	2	Usa	747	25,253	33.81	Shanghai Jiao Tong Univ	56	1,293	23.09
	3	Germany	187	8047	43.03	Capital Med Univ	55	603	10.96
	4	Italy	166	6128	36.92	Southern Med Univ	55	949	17.25
	5	England	129	7027	54.47	Nanjing Med Univ	54	1,298	24.04
	6	Canada	111	5385	48.51	Cent South Univ	43	725	16.86
	7	France	108	4301	39.82	Harvard Med Sch	42	1,342	31.95
	8	Australia	83	3433	41.36	Huazhong Univ Sci & Technol	41	3,548	86.54
	9	India	73	1971	27.00	Fudan Univ	36	503	13.97
	10	Japan	72	2121	29.46	Mayo Clin	36	1,883	52.31

The top 10 research institutions were affiliated with China and the United States (Table 1), among which 80% were located in China.^[23]^ The top 5 institutions by publication output were Wuhan University (70 publications), Shanghai Jiao Tong University (56 publications), Capital Medical University (55 publications), Southern Medical University (55 publications), and Nanjing Medical University (54 publications). Visualization of the collaborative network among 3846 institutions (Fig. 2B) revealed the following patterns: Chinese universities represented by Wuhan University and Shanghai Jiao Tong University formed a green core cluster with dense internal connections, indicating frequent domestic collaborations; red nodes including Harvard Medical School and Mayo Clinic in the United States occupied key positions in the network and maintained close cooperation with Chinese universities; institutions from other countries such as the University of Toronto and the University of Melbourne appeared as purple nodes with relatively weak relevance to the core network.^[24]^ Overall, the collaborative pattern was characterized as “Chinese universities as the core, close interaction between Chinese and American institutions, and auxiliary participation of multi-country institutions.”

### 3.3. Author analysis

The top 10 authors in terms of publication output are listed in Table 2 (Left). A total of 16,835 authors participated in relevant research in this field. Among them, Yang Yang ranked first with 27 publications, 339 TC, and 12.56 citations per paper, followed by Bellomo Rinaldo (19 publications, 1265 TC, 66.58 citations per paper), Yang Wenwen (15 publications, 174 TC, 11.60 citations per paper), and Vincent Jean-Louis (14 publications, 343 TC, 24.43 citations per paper).^[25]^ Notably, Ronco Claudio, who ranked fifth in publication output, topped the list with 117.23 citations per paper. Among the top 10 authors, 6 were from China, two from Italy, one from the United States, and one from Belgium.

**Table 2:** Top 10 authors and co-cited authors related to septic myocardial injury.

Type	Rank	Author	Publications	TC	TC/Publication	Rank	Co-cited author	TC	Total link strength
Septic myocardial injury	1	Yang, Yang	27	339	12.56	1	Singer, M	514	8903
	2	Bellomo, Rinaldo	19	1265	66.58	2	Vincent, Jl	371	6504
	3	Yang, Wenwen	15	174	11.60	3	Li, N	221	4520
	4	Vincent, Jean-louis	14	342	24.43	4	Hollenberg, Sm	215	4158
	5	Deng, Chao	13	166	12.77	5	Wang, Y	205	4054
	6	Ronco, Claudio	13	1524	117.23	6	Bellomo, R	202	4503
	7	Wang, Jian	13	128	9.85	7	Hotchkiss, Rs	200	4139
	8	Chen, Ying	12	95	7.92	8	Zhou, H	186	2907
	9	Kellum, John A.	12	1528	127.33	9	Rhodes, A	180	3215
	10	Lei, Wangrui	12	147	12.25	10	Rudiger, A	179	3527

Table 2 (Right) presents the ranking of co-cited authors, which clearly depicts the influence landscape of core scholars in the field. Singer M and Vincent JL ranked among the top with high TC and total link strength, establishing themselves as internationally authoritative scholars. The ranking includes Chinese scholars such as Li N and Wang Y, as well as international scholars, reflecting that Chinese and foreign scholars jointly form the core influence group.^[26]^ Although some scholars, including Bellomo R, had relatively lower TC, they possessed higher total link strength, indicating stronger academic connectivity. On the whole, the field is characterized by “leading international authorities, collaborative participation of Chinese and foreign scholars, and influence combining citation popularity with academic relevance. The co-authorship network map in Figure 2C shows that multiple color-coded collaborative clusters have formed in the field. Within each cluster, core nodes such as Yang Yang and Yang Wenwen maintained close intra-team collaboration; however, connections between different clusters were sparse, indicating low relevance of inter-team cooperation.^[27]^ Overall, the collaborative pattern is characterized as “parallel research by multiple teams, tight intra-team collaboration, and limited inter-team interaction.

### 3.4. Journal analysis

In terms of journal publication distribution in the field of sepsis-induced myocardial injury, the landscape of the top 10 journals by publication output is clearly defined (see Table 3 and Figure 2D): Shock ranked first with 50 publications (accounting for 1.71%), establishing itself as one of the core venues for publishing research findings in this field. It was closely followed by Critical Care, Critical Care Medicine, and PLOS ONE, each with 42 publications (1.43% of the total), which together formed the second tier of journals by output volume. Frontiers in Immunology ranked fifth, with 38 publications (1.3%).^[28]^ From the perspective of academic influence, the scholarly value of journals exhibited the characteristic of “publication output being incompletely correlated with citation popularity. Among the top 10 journals by publication volume, Critical Care not only contributed 42 publications but also led the field with 2230 TC, with an average of 53.10 citations per paper, making it the most impactful journal in this tier for knowledge dissemination. Although Intensive Care Medicine published only 12 relevant articles, it received 1699 citations, demonstrating a high-impact “quality over quantity” trait. Particularly notable was Cellular Physiology and Biochemistry: with a mere 3 publications, it accumulated 1122 TC, and its average citations per paper hit 374, ranking first among all journals and emerging as a representative journal with “extremely high per-article influence” in this field.^[29]^ This distribution pattern not only reflects that journals such as Shock and Critical Care are the mainstream publishing platforms for sepsis-induced myocardial injury research, but also indicates that some journals have achieved core influence through high-quality outputs characterized by “low publication volume yet high citation rates.^[30]^

**Table 3:** Top 10 journals and co-occurrence network related to septic myocardial injury.

Type	Rank	Journals	Publications	TC	TC/Publication	Key	TC	Total link strength
Septic myocardial injury	1	Shock	50	829	16.58	Sepsis	1278	9428
	2	Critical care	42	2230	53.10	Mortality	506	3842
	3	Critical care Medicine	42	2184	52.00	Septic shock	476	3978
	4	Plos One	42	1307	31.12	Acute kidney injury	443	3563
	5	Frontiers in Immunology	38	1096	28.84	Inflammation	414	3210
	6	Scientific reports	38	966	25.42	Injury	345	2650
	7	Cureus Journal Of Medical Science	37	127	3.43	Dysfunction	306	2353
	8	Journal of Clinical Medicine	37	486	13.14	Apoptosis	288	2324
	9	International immunopharmacology	36	406	11.28	Oxidative stress	237	1955
	10	Frontiers in pharmacology	31	605	19.52	Myocardial injury	229	1559

### 3.5. Article analysis

The distribution characteristics and research landscape of the top 10 most-cited articles (Table 4) in the field of sepsis- induced myocardial injury clearly outline the core development trajectory and academic priorities of this discipline: all the top 10 articles have accumulated over 586 citations, demonstrating remarkable academic influence. Among them, the study by Chen et al (2020), published in BMJ (IF = 40.3) and focusing on the clinical characteristics of COVID-19 fatal cases, ranked first with 3230 citations. This finding highlights the high research attention paid to sepsis-associated multiple organ dysfunction (including myocardial injury) in the context of public health emergencies. In addition, the works by Kellum et al (2021) and Cohen et al (2015) were published in top-tier journals such as Nature Reviews Disease Primers (IF = 60.6) and Nature Reviews Drug Discovery (IF = 101.8), reflecting the close integration of high-quality research in this field with internationally authoritative journals. The research topics present a diversified layout characterized as “basic mechanisms - clinical applications - cross-disciplinary correlations.”^[31]^ They not only include the exploration of core mechanisms, such as the proposition by Li et al (2020) that “ferritinophagy-mediated ferroptosis is involved in sepsis-induced cardiac injury, but also cover interrelated fields and key clinical issues, including acute kidney injury, ischemia-reperfusion injury, cytokine storm intervention, and clinical blood transfusion strategies. This reflects the in-depth integration of sepsis-induced myocardial injury research with multiple organ dysfunction, fundamental pathological mechanisms, and clinical practice.^[32]^ Meanwhile, the value of these publications exhibits the feature of “incomplete positive correlation between IF and citation frequency.” For instance, the article by Wu et al (2018), published in Cellular Physiology and Biochemistry (IF = 2) and exploring the mechanisms of ischemia-reperfusion injury, ranked third with 1266 citations, demonstrating enduring academic value. Furthermore, core findings from both Chinese and foreign scholars have been included in the top 10 list: Chinese research teams have not only participated in clinical characteristic analysis but also devoted themselves to mechanism exploration, reflecting a collaborative pattern in which Chinese and foreign scholars jointly lead the development of this field.^[33]^ Overall, these 10 highly cited articles collectively illustrate the current research status and development trends of the sepsis-induced myocardial injury field, which are characterized by “interdisciplinary integration, equal emphasis on basic and clinical research, and high-impact outcomes focusing on core mechanisms and practical demands.

**Table 4:** Top 10 highly cited paper related to septic myocardial injury.

Type	Rank	Authors	Article title	Source title	Times cited, all databases	IF	DOI link
Septic myocardial injury	1	Chen (2020)	Clinical characteristics of 113 deceased patients with coronavirus disease 2019: retrospective study	BMJ-BRITISH MEDICAL JOURNAL	3230	40.3	http://dx.doi.org/10.1136/bmj.m1091
	2	Kellum (2021)	Acute kidney injury	NATURE REVIEWS DISEASE PRIMERS	1359	60.6	http://dx.doi.org/10.1038/s41572-021-00284-z
	3	Wu (2018)	Current mechanistic concepts in ischemia and reperfusion injury	CELLULAR PHYSIOLOGY AND BIOCHEMISTRY	1266	2	http://dx.doi.org/10.1159/000489241
	4	Cohen (2015)	Muscle wasting in disease: molecular mechanisms and promising therapies	NATURE REVIEWS DRUG DISCOVERY	999	101.8	http://dx.doi.org/10.1038/nrd4467
	5	Fang (2023)	The molecular and metabolic landscape of iron and ferroptosis in cardiovascular disease	NATURE REVIEWS CARDIOLOGY	858	44.2	http://dx.doi.org/10.1038/s41569-022-00735-4
	6	Murphy (2015)	Liberal or restrictive transfusion after cardiac surgery	NEW ENGLAND JOURNAL OF MEDICINE	680	78.5	http://dx.doi.org/10.1056/NEJMoa1403612
	7	Sun (2015)	Association of intraoperative hypotension with acute kidney injury after elective noncardiac surgery	ANESTHESIOLOGY	657	9.2	http://dx.doi.org/10.1097/ALN.0000000000000765
	8	Wang (2017)	Cardiac surgery-associated acute kidney injury: risk factors, pathophysiology and treatment	NATURE REVIEWS NEPHROLOGY	633	39.8	http://dx.doi.org/10.1038/nrneph.2017.119
	9	Li (2020)	Ferritinophagy-mediated ferroptosis is involved in sepsis-induced cardiac injury	FREE RADICAL BIOLOGY AND MEDICINE	586	8.2	http://dx.doi.org/10.1016/j.freeradbiomed.2020.08.009
	10	Tanaka (2016)	Immunotherapeutic implications of IL-6 blockade for cytokine storm	IMMUNOTHERAPY	629	2.3	http://dx.doi.org/10.2217/imt-2016-0020

**Figure 4.**
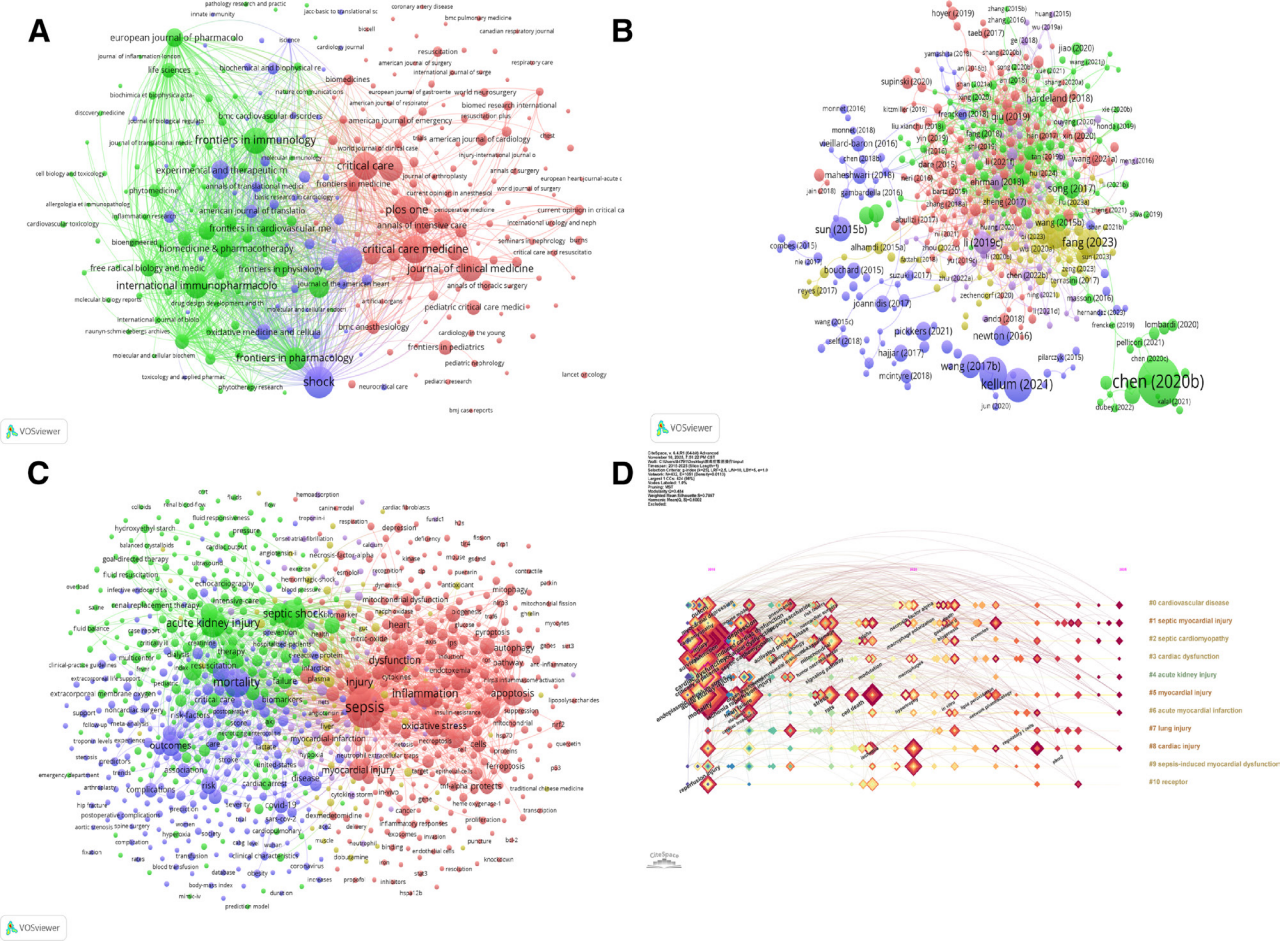
(A) Article-level collaboration network, (B) Keyword-level collaboration network, (C) Keyword timeline cluster map, (D) Top 25 documents with the strongest citation burst.

Figure 4A, as the literature co-citation network map in the field of SICM, clearly presents the literature correlations and research threads of this discipline: nodes of different colors in the map correspond to other articles, and the connections between nodes represent co-citation relationships, thus forming several relatively independent literature clusters (e.g., green, red, and blue clusters). Each cluster corresponds to a set of articles with similar research topics and high co-citation frequencies, indicating that the field has evolved into multiple research branches focusing on different sub-directions. Meanwhile, prominent nodes such as “Chen (2020b)” and “Kellum (2021)” represent the core literature of their respective clusters, indicating that these works are landmark studies across various subfields and enjoy greater citation impact and academic connectivity within the discipline.^[34]^ In addition, the small number of connections between different clusters suggests that various sub-research directions are not entirely isolated but do have certain academic intersections and co-citation relationships.

### 3.6. Keyword analysis

Combined with the keyword ranking in Table 3 (Right), the research hotspots and evolutionary logic in the field of sepsis-induced myocardial injury revealed by Figures 4B and 4C can be elaborated as follows. In the keyword co-occurrence network shown in Figure 4B, Sepsis emerged as the field’s core thematic term, with an occurrence frequency of 1278 and a total link strength of 9428.^[35]^ The cluster formed around it was closely associated with high-frequency keywords, including Mortality (506 occurrences), Septic Shock (476 occurrences), and Acute Kidney Injury (443 occurrences), thereby laying the foundation for the field’s basic research framework. Meanwhile, 3 primary sub-research directions have diverged from this core framework: the basic mechanism cluster centered on Inflammation (414 occurrences), Oxidative Stress (237 occurrences), and Apoptosis (288 occurrences); the pathological injury cluster focusing on Injury (345 occurrences), Dysfunction (306 occurrences), and Myocardial Injury (229 occurrences); and the clinical outcome cluster linked to Mortality, presenting an overall hotspot pattern characterized as “taking Sepsis as the core, with multiple associated themes advancing in parallel.”^[36]^

The temporal distribution of keywords in Figure 4C demonstrates the evolution of research in this field. In the early stages, high-frequency keywords such as Sepsis, Mortality, and Septic Shock laid the foundation of the discipline. In the middle stage, research delved into mechanism-related keywords such as Inflammation, Oxidative Stress, and Apoptosis. In recent years, both the severity and frequency of Acute Kidney Injury and Myocardial Injury have gradually increased, reflecting a research trend toward the sub-direction of “sepsis-associated multiple organ injury (including myocardial injury).” The combination of these 2 analyses indicates that the field, with Sepsis as its core theme, has developed a diversified research system featuring “basic pathology-associated injury-clinical outcomes. Moreover, the research focus is gradually shifting from generalized sepsis research to in-depth exploration of mechanisms such as Inflammation and Oxidative Stress, as well as subtypes of injury, such as Myocardial Injury.^[37]^

### 3.7. Citation burst analysis

Figure 4D presents the statistical results for the top 25 articles with citation bursts in the field of sepsis-induced myocardial injury, clearly illustrating the citation-burst characteristics and temporal distributions of the key findings in this discipline. In the figure, Year Strength represents the intensity of citation bursts, while Begin/End indicates the start and end years of the burst periods.^[38]^ The data show that the citation bursts of most articles occurred between 2015 and 2020. Notably, 2015 emerged as the focal point for multiple articles with high burst intensities (e.g., some articles reached 12.13), suggesting that this period was a critical stage when research interest in the field rose rapidly, and core findings emerged in bursts. These high-intensity burst articles mainly reported breakthrough achievements, such as the proposal of novel mechanisms (e.g., the association between ferroptosis and sepsis-induced myocardial injury), innovative diagnostic and treatment strategies, or studies linked to major public health events, thereby attracting extensive attention and citations from the academic community within a short period.^[39]^

Meanwhile, the citation bursts of some articles have persisted through 2023, reflecting the enduring academic value of these core findings, which remain essential foundations and key references for current research in the field. Overall, this analysis provides a crucial basis for identifying the key developmental nodes, breakthrough achievements, and long-influential literature in the field of sepsis-induced myocardial injury.

## 4. Discussion

### 4.1. Basic information

This study highlights the current state of research and cutting-edge directions in global SICM over the past decade. A total of 2927 publications retrieved from the WoSCC were analyzed using VOSviewer, CiteSpace, and Bibliometrix. The results demonstrated that the evolution of this field, as reflected in trends in annual publications and citations, has undergone 3 distinct phases: steady accumulation, explosive growth, and mature stabilization.^[40]^ The accumulation phase from 2015 to 2019 laid a solid academic foundation for the field’s development. The explosive growth in 2020 propelled the discipline into rapid advancement, and the stable development since 2021 has marked the maturation of the research system in this field. Currently, the field remains in an active research state; the phased fluctuations in the 2025 data do not undermine the overall positive development trend.^[41]^ As research deepens, it is expected that more high-quality findings will be generated, further enhancing the academic influence and translational clinical value of this domain.

SICM research has formed a global landscape, with China occupying a core position in this field. China ranks first worldwide in both the number of publications (1212 articles) and TC (9331), emerging as a crucial driving force in advancing research in this discipline. However, it should be noted that China’s average citations per paper (21.55) rank relatively low, placing it tenth among the top 10 countries by publication volume.^[42]^ In contrast, countries such as the United Kingdom, Canada, and Germany exhibit significantly higher average citations per paper, suggesting that China needs to improve the quality of its research outputs further. Collaborative network analysis revealed that the United States, China, and European countries constitute the core entities with the highest levels of cooperation. Among them, the United States and China maintain broader collaborative scopes covering multiple continents. Domestically, institutions have established close collaborations, forming a China-based university-led cooperative cluster. Cross-regional cooperation between Chinese and American institutions is also closely linked, presenting an overall pattern characterized by “Chinese universities as the core, tight interaction between China and the United States, and auxiliary participation of multiple countries.” In terms of institutional contributions, 4-fifths of the top 10 high-yield institutions are from China; 5 Chinese universities, including Wuhan University, Shanghai Jiao Tong University, and Capital Medical University, rank among the top 5 institutions in publication volume.^[43]^ At the author level, Chinese scholar Yang Yang leads in publication output with 27 articles. In contrast, Ronco Claudio, despite ranking fifth in publication volume, tops the list in average citations per paper. The core influential group in this field comprises both Chinese and foreign scholars: Singer M and Vincent, J.L. are recognized as leading international authorities. Meanwhile, the field also exhibits the characteristic that “publication volume is not completely correlated with citation popularity. Regarding journal distribution, Shock has the highest publication output and serves as a core venue for disseminating research findings in this field.^[44]^ In contrast, Cellular Physiology and Biochemistry, which has published only 3 relevant articles, ranks first among all journals, with an average of 374 citations per paper. Intensive Care Medicine also demonstrates the high-impact trait of “quality over quantity, indicating that the academic value of a journal should be evaluated by both publication volume and the quality of its outputs. The core research directions of this field revolve around Sepsis, forming a diversified system featuring “basic pathology - associated injury - clinical outcomes.”^[45]^ The research focus is gradually shifting from generalized sepsis research to in-depth exploration of mechanisms such as inflammation and oxidative stress, as well as a specialized focus on myocardial injury. Highly cited articles focus on core mechanisms and clinical practice needs, with Chinese and international scholars collaborating to advance this field.

Keyword clustering analysis indicated that the high-frequency core keywords are sepsis (1278 occurrences), mortality (506 occurrences), and septic shock (476 occurrences). The research directions center on septic myocardial injury, septic cardiomyopathy, and cardiac dysfunction, covering cardiovascular diseases, multiple organ complications (e.g., acute kidney injury, lung injury), and molecular receptor mechanisms. From a temporal perspective, the research focus has shifted from early basic mechanisms (e.g., cell death, inflammatory response) to specialized areas such as septic cardiomyopathy subtypes, multi-organ interactions, and molecular targets.^[46]^ The high-frequency co-occurrence of core keywords has established a comprehensive research thread spanning from basic mechanisms to clinical subtypes, and from isolated myocardial injury to multisystem associations, intuitively highlighting the field’s research hotspots and development trends.^[47]^ Citation burst analysis showed that these publications are mainly published in authoritative journals in critical care medicine, cardiovascular medicine, and immunology, with research focusing on sepsis, myocardial injury, critical care, and immune mechanisms. In terms of burst period trends, literature in the early stage (2013–2016) focused on the diagnosis and treatment of sepsis and the basic mechanisms of myocardial injury; research in the middle stage (2017–2020) delved into specialized directions such as immune regulation and multi-organ interactions; and recent literature (2021–2025) has extended to the association between aging, cachexia, and myocardial injury. This analysis directly reflects the dynamic changes in research hotspots and the continuous transmission of academic influence in this field. In addition, cross-regional collaboration and interdisciplinary integration are essential characteristics of the field’s development.^[48]^ The high attention received by the China-US-Europe core cooperation circle and multidisciplinary research themes confirms that cross-regional and interdisciplinary collaboration plays a vital role in advancing SICM research.

### 4.2. Research hotspots and frontiers

Research on SICM has attracted sustained attention from scholars worldwide. A combined analysis of keywords, citation bursts, and cutting-edge advances indicates that current research frontiers in this field are focused on mitochondrial metabolic reprogramming, epigenetic regulation (e.g., m^6^A methylation, noncoding RNAs), immune-metabolic crosstalk, and the development of specific biomarkers. In addition, cross-regional collaboration and interdisciplinary integration have emerged as crucial drivers of this field’s development.^[49]^ The formation of the China-US-Europe core cooperation network and the in-depth integration of multiple disciplines, including critical care medicine, cardiovascular medicine, and immunology, have continuously propelled the research on SICM from basic mechanism exploration toward precision diagnosis and targeted therapy.

### 4.3. Clinical practice and diagnosis

Sepsis is a life-threatening heterogeneous syndrome characterized by dysregulated host responses to infection that lead to organ dysfunction, and it ranks among the major diseases posing a severe threat to human health worldwide. Research on SICM has thus become increasingly important. The 2025 Chinese Expert Consensus on Emergency Medicine has clearly defined the concepts of SICM and septic-related cardiomyopathy (SRCD): SICM is primarily diagnosed based on elevated cardiac troponin levels with the exclusion of acute myocardial infarction; SRCD is identified by a left ventricular ejection fraction <50%, abnormal global longitudinal strain, and elevated B-type natriuretic peptide (BNP)/N-terminal pro-BNP (NT-proBNP) levels, accompanied by hypoperfusion, with echocardiography as the core assessment tool. This consensus distinguishes SICM and SRCD as distinct subtypes, clarifies their diagnostic criteria, and establishes echocardiography as the gold standard for evaluation.^[50]^ This refined definition fills the gap in the field’s diagnostic criteria, providing a unified basis for the precise clinical identification and stratified management of sepsis patients with cardiac involvement. Meanwhile, it lays a foundation for the accurate delineation of research cohorts in scientific studies, thereby helping reduce outcome bias and data heterogeneity caused by inconsistent definitions across studies.

SICM are critical conditions associated with high mortality in the intensive care unit. From an epidemiological perspective, the global incidence of SICM ranges from 10% to 70%.^[51]^ Notably, patients complicated with SRCD exhibit poor prognostic features, including prolonged ICU length of stay and significantly increased mortality. The substantial variation in reported incidence rates stems from differences in diagnostic criteria, baseline characteristics of study populations, and research methodologies (Zhu GJ et al 2025). These epidemiological data not only highlight the high prevalence and severe harm of this condition among sepsis patients, but also suggest that early interventions for such high-risk populations should be prioritized in clinical practice.^[52]^ Furthermore, clarifying the factors contributing to the heterogeneous incidence rates guides the conduct of future multicenter, standardized epidemiological studies, thereby facilitating a more accurate assessment of the global disease burden of SICM.

### 4.4. Mitochondrial dysfunction and metabolic reprogramming

In recent years, sepsis has been shown to induce myocardial injury and dysfunction, resulting in persistently high mortality rates among affected patients. Myocardial cell apoptosis positively regulates the development of septic myocardial injury and dysfunction. Studies have demonstrated that during sepsis, the lactylation of key enzymes, such as HADHA, inhibits fatty acid oxidation, leading to decreased ATP production and impaired myocardial contractility (Gekle M et al 2024). Additionally, TGF-β3 regulates metabolic switching via Smad7, while AMPK dysregulation is associated with inflammation and abnormal mitochondrial quality control (Hollenberg SM et al 2021). The development of specific inhibitors targeting HADHA lactylation, as well as targeted modulation of the TGF-β3/Smad7 pathway or AMPK activity, is expected to address the current lack of specific therapeutic agents for septic myocardial injury, thereby providing core targets for the development of precision-targeted drugs. Meanwhile, these key molecules (e.g., lactylated HADHA, TGF-β3, AMPK) can also serve as potential biomarkers for the early diagnosis or prognostic evaluation of septic myocardial injury, facilitating precise stratified management in clinical practice.

### 4.5. Epigenetic and noncoding RNA regulation

Currently, a growing body of evidence has linked N^6^-methyladenosine (m^6^A) modification and ferroptosis to sepsis-induced myocardial injury. Studies have found that m^6^A methylation (mediated by regulators such as METTL3) coordinates inflammation, apoptosis, and ferroptosis in sepsis-induced myocardial injury (Wang X et al 2025). Inflammatory storm and programmed cell death (apoptosis and ferroptosis) are core pathological processes of sepsis-induced myocardial injury. As the most prevalent epigenetic modification of eukaryotic mRNA, m^6^A methylation can simultaneously regulate the expression of key genes involved in the above processes through transcriptome remodeling.^[53]^ This finding clarifies the central role of epigenetic regulation in sepsis-induced myocardial injury and also explains the molecular basis for the synergistic activation of multiple pathological pathways during sepsis. From an intervention perspective, targeting m^6^A methylation regulators (e.g., inhibiting METTL3 activity) or their downstream key pathways is expected to achieve synchronous regulation of inflammation, apoptosis, and ferroptosis, providing a novel direction for the development of multi-target synergistic intervention strategies. This approach aligns with the clinical demand of addressing the complex pathological mechanisms of sepsis-induced myocardial injury, where single-target interventions yield limited efficacy.

Studies have demonstrated that platelet-derived extracellular vesicles carry miR-885-5p to mediate myocardial injury via the HMBOX1/NF-κB/NLRP3 pathway. Inhibition of EV biogenesis by GW4869 can ameliorate cardiac function (Liao B et al 2025). Elucidation of this mechanism provides direct evidence for deciphering the transmission pathway of systemic inflammation to local myocardial injury in sepsis, thus improving the pathological chain of “systemic inflammation - intercellular communication - local myocardial injury.”^[54]^ On the other hand, the therapeutic effect of GW4869, an EV biogenesis inhibitor, confirms that targeting EV biogenesis or the signaling pathways they mediate is cardioprotective. Moreover, GW4869 has been used in multiple basic research studies and has potential translational clinical value, offering a directly exploitable direction for the development of targeted therapeutic agents.

The discovery that exosomal miR-150-5p and miR-155 can serve as diagnostic biomarkers for sepsis-induced myocardial injury is consistent with the aforementioned potential biomarkers, including HADHA lactylation, TGF-β3, and AMPK. Collectively, these findings support the development of a precise diagnostic system for sepsis-induced myocardial injury (Liao B et al 2025), further enriching the repertoire of accurate diagnoses and treatment targets for this condition. Together with strategies such as targeting HADHA lactylation modification, the TGF-β3/Smad7 pathway, and AMPK activity, these biomarkers constitute a multi-dimensional, precise intervention system characterized by “epigenetic regulation - intercellular communication intervention - energy metabolism modulation.^[55]^

### 4.6. Cell death and immune-metabolic crosstalk

Ferroptosis, an iron-dependent novel form of programmed cell death, has been confirmed to play a central role in SICM. This process clearly involves 3 core pathways: the GSH/GPX4 pathway, iron metabolism, and lipid metabolism. Furthermore, the potential target value of the FSP1-CoQ10-NAD(P)H system provides a specific direction for intervention strategies targeting ferroptosis (Wang X et al 2025).

The imbalance mechanism of the inflammation-immunity-metabolism network, particularly the excessive activation of NF-κB and the NLRP3 inflammasome, and the impact of macrophage M1/M2 polarization on injury progression, reveals the “systemic-local” synergistic regulatory logic of SICM (Zou XZ et al 2023). This finding breaks the traditional view of “inflammation as the sole pathogenic factor,” clarifies the synergistic injury cascade of “inflammatory activation - abnormal immune cell polarization - metabolic disorder,” and explains the core reasons for the protracted course and poor repair of SICM.

Meanwhile, the reparative effect of macrophage M2 polarization also provides a new direction for intervention. Inducing macrophage polarization toward the M2 phenotype by regulating key molecules such as Hmgcs2 can achieve a dual effect of “anti-inflammation + tissue repair.”^[56]^ The development of such immunometabolic modulators is expected to provide novel strategies for subsequent repair treatment of SICM, aligning with the clinical demand for myocardial function recovery after injury.

### 4.7. Precision diagnosis and biomarkers

The hierarchical application of classical biomarkers (cTn, BNP/NT-proBNP, h-FABP) and novel biomarkers (miR-378a-3p, miR-21-3p, LCN, HO-1, etc) represents a landmark advance in constructing a precision diagnosis and treatment system for SICM, while providing dual support for clinical management and scientific research.^[57]^ Among these findings, the conclusion that cTn and BNP/NT-proBNP are applicable for prognostic evaluation, whereas h-FABP holds higher diagnostic value, clarifies the differentiated application scenarios of classical biomarkers and addresses the core clinical challenges of SICM, namely difficulty in early identification and ambiguity in prognostic judgment (Frencken JF et al 2018).

Compared with classical biomarkers, noncoding RNAs such as miR-378a-3p and miR-21-3p exhibit strong tissue specificity and high stability, enabling them to reflect the molecular pathological processes of SICM accurately. In contrast, protein biomarkers, including LCN and HO-1, can correlate synchronously with core pathological processes such as inflammatory responses and oxidative stress, thereby improving diagnostic specificity and providing refined evidence for stratifying disease severity (Xie Y et al 2025). The discovery of these novel biomarkers has also facilitated the in-depth elucidation of SICM’s pathological mechanisms. Through the molecular pathways associated with these biomarkers, potential pathogenic targets can be further explored, thus forming a closed research loop characterized by “biomarkers - mechanisms - interventions.

### 4.8. Exploration of therapeutic targets

At present, clinical treatment for SICM is mainly symptomatic and supportive, lacking specific targeted drugs; moreover, single-target intervention is difficult to address the synergistic damage caused by its complex multi-pathological processes. In contrast, active components of traditional Chinese medicine (TCM), such as astragalus polysaccharide and ginsenoside, possess inherent multitarget regulatory properties (Zou XZ et al 2023). Activation of the AMPK pathway via these components can simultaneously exert 3 core effects: first, inhibiting excessive inflammatory responses and alleviating myocardial damage induced by inflammatory storms; second, ameliorating metabolic disorders in cardiomyocytes and reversing the abnormal shift from fatty acid oxidation to glycolysis during sepsis; third, repairing mitochondrial function and restoring myocardial energy supply. This mode of action, characterized by “multi-pathway synergistic regulation,” perfectly aligns with the clinical demand arising from the complex pathological mechanisms of SICM, overcoming the limitations of traditional chemical drugs that rely on single-target intervention. Meanwhile, as natural products, TCM active components have high safety profiles, making them more amenable to clinical translation compared with some chemical inhibitors. They thus provide promising candidates for the development of effective and safe therapeutic agents for SICM, as well as robust experimental support for modernizing TCM and integrating TCM and Western medicine in the treatment of critical illnesses.^[58]^

The cardioprotective effects of immunometabolic modulators (e.g., m^6^A inhibitors, NF-κB inhibitors) demonstrated in animal experiments have further improved the precision-targetedintervention system for SICM, offering crucial support for resolving therapeutic dilemmas at the molecular mechanism level (Wang X et al 2025). m^6^A inhibitors can synchronously block the abnormal activation of multiple pathological processes by regulating epigenetic modifications; NF-κB inhibitors, on the other hand, can directly suppress inflammatory signaling pathways and cut off the core chain of inflammatory damage. The development of such modulators marks the upgrading of SICM intervention strategies from “broad-spectrum anti-inflammation and symptomatic support” to “precision targeting of molecular mechanisms, enabling the realization of individualized treatment. In addition, the confirmed protective effects in animal experiments have laid a solid foundation for subsequent preclinical studies and clinical trials, accelerating the translation of basic mechanism research into clinical therapy.

## 5. Strengths and limitations

In this study, a bibliometric visualization analysis method was used to explore the association between sepsis and myocardial injury systematically. We comprehensively reviewed the developmental context and evolutionary patterns of this field, providing a structured research reference for the academic community while clearly defining the current state of research, key hotspots, and future development trends.

Nevertheless, this study has certain limitations. First, the literature retrieval was based solely on the Web of Science (WOS) database, potentially leading to the omission of relevant publications in other databases and introducing database bias. Second, the included literature was restricted to English articles published between 2015 and 2025 and indexed in the WoSCC. This selection criterion may have led to the exclusion of high-quality studies in other languages or databases. Third, some recently published high-quality articles are difficult to include in the top 10 journals and top 10 most-cited publications lists due to their low citation counts. Meanwhile, bibliometric analysis relies on citation indicators. It cannot fully assess the intrinsic academic quality of individual studies, and the low citation frequency of newly published studies may affect the accuracy of their interpretation. Fourth, differences in the classification of subject terms and the annotation of keywords across articles may introduce bias into the analysis. Despite the aforementioned limitations, the application of visualization methods to analyze the research status, hotspots, and development trends in this field remains a valuable academic reference.

## 6. Conclusion

Over the past decade, research on SICM has experienced rapid development and maintained a high-level of activity. China has demonstrated outstanding research strength in this field, with Wuhan University, Shanghai Jiao Tong University, Capital Medical University, and Southern Medical University emerging as major contributors to studies on SICM. Yang Yang and Rinald Bellomo are the key contributors driving advancements in this domain.

Shock is one of the core journals for publishing research findings in this field. In contrast, journals such as Critical Care, Critical Care Medicine, and PLOS One have also made remarkable contributions. Core research hotspots revolve around septic myocardial injury and septic cardiomyopathy, with studies now advancing to the molecular mechanism level. Mitochondrial metabolism, epigenetics, and regulation of cell death have been identified as key therapeutic targets.

Moving forward, it is imperative to strengthen translational medicine research, drive breakthroughs in precision diagnosis and targeted therapy, and reduce sepsis-related cardiac mortality. These efforts hold profound clinical and scientific significance.

## Acknowledgments

The summary datasets for the Mechanisms of Core Research Hotspots in Sepsis-induced Myocardial Injury from the WoS Core Collection database. We want to express our sincere appreciation to all the contributors and researchers involved in the WoS Core Collection database for their invaluable participation.

## Author contributions

**Conceptualization:** Zhikang Mei.

**Data curation:** Zhikang Mei, Zhirui Zhang.

**Investigation:** Zhikang Mei, Menglu Zhang, Zhirui Zhang, Hong Jiang.

**Project administration:** Zhikang Mei, Tao Hao, Hong Jiang.

**Resources:** Zhikang Mei, Menglu Zhang, Tao Hao, Hong Jiang.

**Validation:** Zhikang Mei, Menglu Zhang, Tao Hao, Hong Jiang.

**Formal analysis:** Menglu Zhang.

**Funding acquisition:** Menglu Zhang, Hong Jiang.

**Software:** Menglu Zhang, Tao Hao, Zhirui Zhang.

**Supervision:** Menglu Zhang, Tao Hao.

**Visualization:** Tao Hao, Zhirui Zhang.

**Methodology:** Zhirui Zhang, Hong Jiang.

**Writing – original draft:** Zhikang Mei.

**Writing – review & editing:** Tao Hao, Hong Jiang.
